# Aerodynamic efficiency assessment of a cross-axis wind turbine integrated with an offshore deflector

**DOI:** 10.1016/j.heliyon.2024.e36412

**Published:** 2024-08-22

**Authors:** Sam Saham, Saber Rezaey

**Affiliations:** Faculty of Mechanical Engineering, Tarbiat Modares University, Tehran, Iran

**Keywords:** Cross axis wind turbine, Offshore CAWT, Flow deflector, DMST, BEM

## Abstract

The present work examines the performance of an offshore cross-axis wind turbine (CAWT) with a flow deflector by integrating numerical and analytical methods. The deflector's geometry redirects flow in all directions, causing it to exit vertically and collide with the wind turbine's horizontal blades. In contrast, the blades of a vertical axis wind turbine (VAWT) harness the power of horizontal wind flow. The total power absorbed by the horizontal and vertical turbine blades represents the power of CAWT. In this study, the speed of the outflow from the deflector was initially determined through numerical simulation. The numerical simulation output was then utilized as an input for analytical Double Multiple Stream Tube (DMST) and Blade Element Momentum (BEM) methods to evaluate the vertical and horizontal turbine blades, respectively. This approach reduces the overall simulation time and establishes an offline coupling between analytical and numerical approaches. The findings of this research have unveiled a promising future for offshore wind energy generation. Through the implementation of a modeled deflector on a Cross-Axis Wind Turbine (CAWT), the power output reached a remarkable 19 KW with a power coefficient of 0.35 at an 8.4 m/s wind speed. The results indicate that the CAWT with the deflector produced a power output 35 % higher and was 45 % more efficient than a single Vertical-Axis Wind Turbine (VAWT). These outcomes illustrate the potential for greater energy production and efficiency in offshore wind farms.

## 1 Introduction

### 1.1 Background of wind energy

**Fig. 1:**
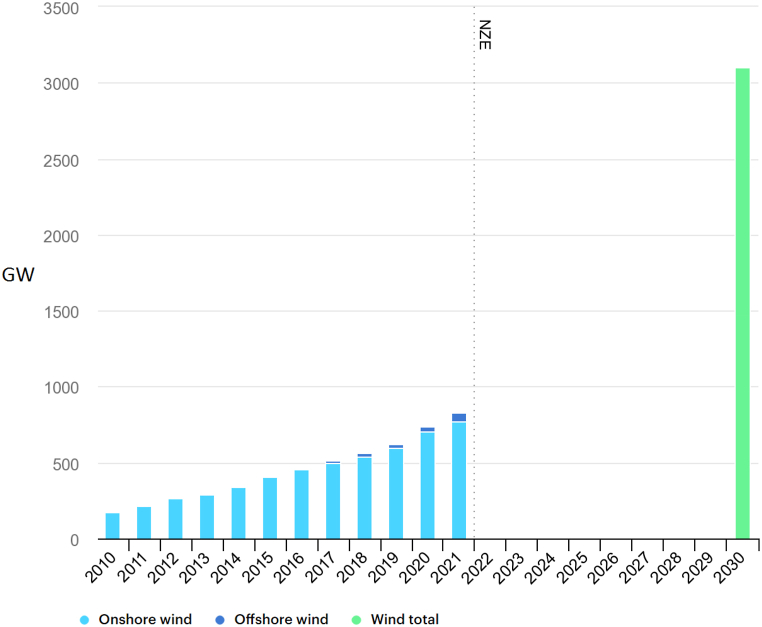
The global cumulative capacity of wind energy [3].

The use of fossil fuels has been linked to various environmental issues, including water pollution, greenhouse gas emissions, and contamination of crops [1]. However, as oil and gas reserves continue to deplete while industrial energy demands continue to rise, many countries are turning to renewable energy sources such as wind and solar power. This type of energy is commonly referred to as green energy and currently accounts for over 20 % of the world's energy needs [2]. Wind energy is one of the most cost-effective and readily available sources of green energy. While onshore wind farms have been well-established, the majority of the earth's surface is covered by water, and thus, offshore wind farms have the potential to generate more electricity. Despite being 1.5 to 2 times more expensive than onshore wind energy, offshore wind farms are more efficient due to higher wind speeds, consistent wind direction, lower turbulence intensity, and smaller shear layers. These factors allow for reduced turbine tower heights above the water surface. Various types of airfoils are utilized in the construction of wind turbine blades. These aerodynamic objects are designed to have a high lift coefficient and a low drag coefficient. In some wind turbines, symmetrical airfoils, such as the NACA 0021, are employed to reduce costs and facilitate blade manufacturing. According to the International Energy Agency's (IEA) annual report in 2021, of the total 830 GW of wind capacity installed, 93 % were onshore systems, and the remaining were offshore wind farms (Fig. 1). Moreover, the growth rate of offshore technology in 2021 was three times the average of the last 5 years. It is projected that wind energy usage will exceed 3000 GW by 2030 [3].

In 2013, Shires [4] examined the DMST approach and verified its effectiveness by applying it to both H-shaped and Phi-shaped turbines. Modifications to the approach allowed for the examination of advanced blade geometry, including that found in offshore V-shaped turbines. In the same year, Soraghan et al. [5] investigated the effects of lift to drag ratio, solidity, and conical angle on the blade for both H-shaped and V-shaped turbines. In 2016, Attia et al. [6] studied the multi-stage vertical axis wind turbine with straight blades and found that they may function more efficiently than general turbines at greater tip speed ratios. To enhance wind turbine performance, Zamani et al. [7] proposed the J-shape airfoil in 2016. By eliminating the high-pressure surface of the airfoil from the thickest point to the trailing edge, they reduced friction on the airfoil pressure surface and enhanced the turbine's ability to start on its own. Finally, in 2017, Sobhani et al. [8] investigated the effects of the blade cavity in vertical axis wind turbines. They examined the size, position, and form of the hollow in various combinations and were able to improve the turbine's aerodynamic performance.

In 2019, Moghimi and Motawej [9] used the DMST method to examine the aerodynamic efficiency of H-type and Gorlov turbines. They analyzed the impact of various geometric features, including chord, aspect ratio, helix angle, airfoil type, and number of blades, as well as wind speed. Their findings indicated that self-starting turbines performed better with wider chords, thicker airfoils, and higher wind speeds. In 2021, Saham and Karimian [10] conducted a study on a wind turbine with vertical axis that featured angled straight blades. They attempted to redirect some of the flow passing through the turbine by altering the blade angle and using it for ventilation.

### 1.2 Innovation in wind turbines

In 2017, Chong et al. [11] introduced a novel wind turbine called a cross-axis wind turbine. Their model included six horizontally arranged blades perpendicular to three vertical blades. Guides were also used to divert and import the flow into the horizontal rotor. Chen et al. [12] investigated the effect of a flow deflector on a vertical axis wind turbine in 2021. They found that adding a deflector can increase the power coefficient by approximately 20 %. The distance between the deflector and the turbine's center was identified as a critical factor in determining the turbine's output power. In the same year, Kvorc and Kozmar [13] studied the installation of wind turbines on tall building rooftops in urban areas. They suggested that optimal positioning of the wind turbines could significantly increase their output power. Chong et al. [14] studied a cross-axis wind turbine at various heights on a rooftop in 2017. They found that the power coefficient of their turbine increased from 0.034 to 0.126. A pitch angle of 10° was also identified as ideal for the horizontal blades. In 2019, Chong et al. [15] studied a unique cross-axis wind turbine design with deflector integration. Their deflector had a simple flat plate shape, and they found that including a deflector could significantly increase the turbine's power and improve its self-starting ability. Wang et al. [16] conducted an experimental analysis and comparison of stable and unsteady flow in a cross-axis wind turbine in 2019. They discovered that increasing flow turbulence under static and dynamic conditions could accelerate the turbine's rotation and increase power extraction.

In 2021, Jyothirmai et al. [17] investigated and simulated the flow around a flow deflector. The deflector increased the mass flow through the turbine, resulting in decreased starting speed and increased output power. Their findings showed that using a deflector angle of approximately 50° can increase flow velocity by about 5.7 %. Prakash et al. [18] conducted an experimental investigation of a cross-axis wind turbine's operation in 2022. Their study found that using a cross-axis wind turbine could double its torque and output, making it more suitable for urban areas compared to vertical-axis wind turbines. The turbine also demonstrated improved early start-up. In the same year, Tarighi et al. [19] conducted a numerical analysis of the performance of a cross-axis wind turbine designed for the climate of the Mazandaran province. The study revealed that the cross-axis wind turbine had greater power output compared to the vertical-axis wind turbine.

### 1.3 Contribution of the present study

**Fig. 2:**
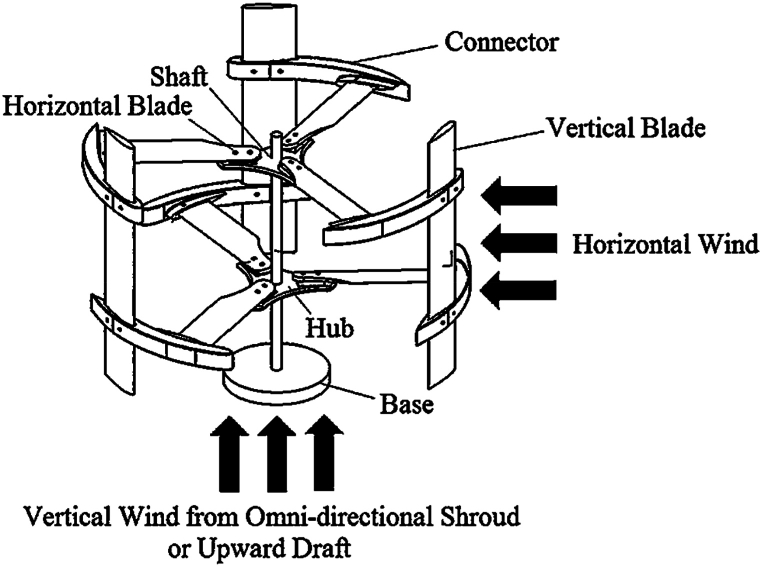
Arrangement of cross axis wind turbine [14].

The conversion of wind energy into mechanical energy is a crucial step in utilizing wind power. Wind turbines accomplish this task, and they can ultimately generate electrical energy through the use of a generator. There are two categories of turbines: horizontal axis and vertical axis. Although vertical axis turbines are less powerful and smaller in size, they are not sensitive to wind direction, and their service costs are lower than one. They are commonly used in residential areas, workshops, on highways, and in offshore locations. The cross-axis wind turbine (CAWT) is a combination of two horizontal axis turbines and two vertical axis turbines. This type of turbine utilizes the benefits of both modern turbines. Fig. 2 shows the turbine's shape, which includes both vertical and horizontal blades. The vertical blade struts are shaped like airfoils to create a horizontal turbine. The dimensions of a CAWT are the same as a vertical-axis wind turbine, but the swept area is larger, resulting in no impact on power production. The CAWT has a better self-starting property than the vertical axis turbine because the Horizontal Axis Wind Turbine HAWT is a self-starting turbine. Table 1 compares the three types of turbines.

**Table 1:** Properties of three types of turbines [20].

Wind turbine type	Wind direction	Self-start	Power coefficient	Methodology
**HAWT**	One direction	Good	0.45	BEM
**VAWT (Lift base)**	All direction	Poor	0.35	DMST
**VAWT (Drag base)**	All direction	Good	0.25	DMST
**CAWT**	All direction	Good	0.5	BEM + DMST

Upon comparison of the power coefficient between the CAWT turbine and two other types, it is evident that the CAWT turbine demonstrates a higher value. This is due to the turbine's ability to benefit from a greater air flow rate, which is made possible by the simultaneous use of two horizontal and vertical axis wind turbines within the CAWT turbine. So, use of two types of wind turbines in the CAWT turbine offers several benefits, including.

1. Higher power output: By using both horizontal and vertical axis wind turbines, the CAWT turbine can capture wind energy from multiple directions, resulting in a higher overall power output compared to a turbine that uses only one type.
2. More efficient energy conversion: The two types of turbines can work together to optimize energy conversion, improving the overall efficiency of the turbine.
3. Increased reliability: The use of two types of turbines can increase the reliability of the CAWT turbine, as it is less likely to experience downtime due to factors such as changes in wind direction or speed.
4. Reduced noise: The use of two types of turbines can help reduce the noise generated by the turbine, as the different types of turbines produce different sound frequencies, which can cancel each other out to some extent.

Overall, using two types of wind turbines in the CAWT turbine can lead to a more efficient and reliable energy generation system.

In light of the growing demand for energy, the cross-axis wind turbine is an effective method of increasing wind turbine efficiency. This study utilizes a CAWT with a flow deflector on the sea surface, where it is referred to as an offshore wind turbine. The wind flows on the water's surface are not turbulent, allowing for some of the horizontal wind to shift vertically before passing through the horizontal rotor. As a result, the turbine's self-starting property and efficiency are expected to improve.

## 2 Baseline geometry

### 2.1 Deflector geometry

**Fig. 3:**
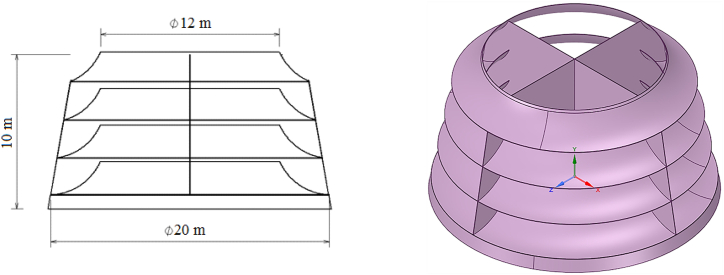
Modeled deflector geometry.

**Fig. 4:**
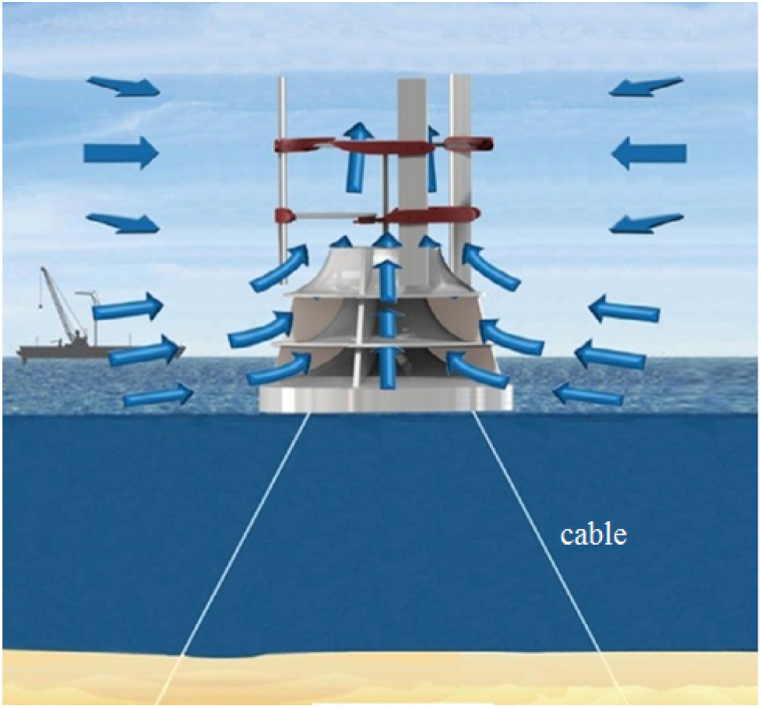
Offshore application of cross axis wind turbine (CAWT) [14].

A deflector is a structure placed on the sea surface that converts horizontal wind flow into a vertical flow. It can also serve as a support for the wind turbine axis. Fig. 3 shows a schematic of this structure. The CAWT is placed on top of the deflector, which is also situated on the sea surface. To prevent it from moving due to waves or wind, cables can connect the deflector to the sea floor, as shown in Fig. 4. If the sea is deep, a suitable foundation can be constructed to stabilize the deflector.

When wind hits the deflector from any direction, it is deflected and escapes from the top center to hit the horizontal blades. The deflector is designed based on the characteristics of Table 2, and its resulting design is shown in Fig. 3.

**Table 2:** Dimension of the deflector.

Parameter	Value
Deflector bottom Diameter (m)	20
Deflector Upper Diameter (m)	12
Deflector total height (m)	10

### 2.2 CAWT geometry

**Fig. 5:**
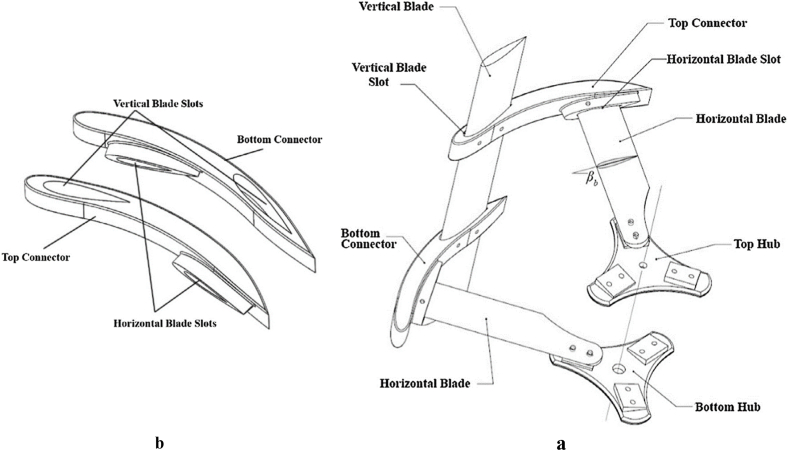
A part of the CAWT rotor. (a) The vertical blade is linked with the horizontal blades via the connectors; (b) The connectors of the CAWT [11].

The NACA airfoils, known for their symmetrical design, have gained popularity among wind turbine researchers. As a result, for the investigation of CAWT [16], the NACA 0015 airfoil was utilized. Fig. 5 displays a general arrangement and detailed view of the Cross-axis wind turbine (CAWT). The figures show a VAWT rotor and two HAWT rotors linked by connectors. These connectors contain two distinct slots for horizontal and vertical turbine blades, while the axle holes connect the HAWT rotor to the hubs. In fact, the HAWT blades are the struts of the VAWT blade, and may or may not have a pitch angle. For the purpose of this study, the pitch angle is set at 5° to enhance the output power.

Additionally, Fig. 2 illustrates the operation of the CAWT. As demonstrated, the side wind (horizontal wind) rotates the VAWT blades, while the vertical wind generated by the deflector interacts with the HAWT blades, generating torque. Fig. 4 depicts the use of a deflector to convert horizontal steady flow on the sea into vertical flow. To suit the turbine in question, three blades are utilized, and their details are listed in Table 3.

**Table 3:** CAWT specification.

Parameter	Vertical blades	Horizontal blades
Airfoil	NACA 0015	NACA 0015
Rotor diameter (m)	15	15
Blade height (m)	15	–
Blade chord (m)	0.5	0.5
Number of blades	3	3
Pitch angle (deg)	0	5
Rotational speed (rpm)	20	20

The CAWT is an omnidirectional turbine, and therefore, does not require yaw control. Furthermore, as depicted in Fig. 2, the CAWT can utilize horizontal blades with vertical wind. This vertical wind flow can be attributed to the deflector or guide beneath the turbine.

## 3 Numerical study

### 3.1 Numerical model

Fluid flow through the deflector is simulated in Fluent software (Version 2019 R3). The governing equations of the fluid in this simulation are.

• Continuity:

(4) $$<listaend>\frac{\partial \rho }{\partial t}+\frac{\partial }{\partial x_{i}}\left(\rho u_{i}\right)=0;\;i=1,2,3$$

• Momentum:

(5) $$\frac{\partial }{\partial t}\left(\rho u_{i}\right)+\frac{\partial }{\partial x_{j}}\left(\rho u_{i}u_{j}\right)=\frac{\partial P}{\partial x_{i}}+\frac{\partial }{\partial x_{j}}\left[\mu \left(\frac{\partial u_{i}}{\partial u_{j}}+\frac{\partial u_{j}}{\partial u_{i}}\right)-\tau_{ij}\right]$$

Where u represents average velocity and $\tau_{ij}$ represents Reynolds stress. Assuming the flow to be incompressible and at steady state, the equations can be expressed as [21]:

(6) $$\frac{\partial }{\partial x_{i}}u_{j}=0$$

(7) $$\frac{\partial }{\partial x_{i}}\left(\rho u_{j}u_{i}-\tau_{ij}\right)=-\frac{\partial P}{\partial x_{i}}$$

**Fig. 6:**
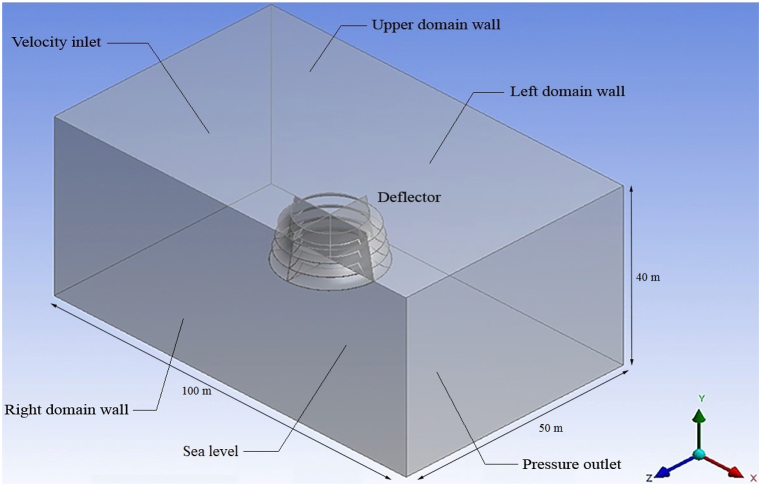
Computational domain and boundary conditions.

The deflector geometry used in the model is complex, causing turbulent airflow. To simulate mean flow characteristics for turbulent flow conditions, the Realizable K-epsilon (k-ε) turbulence model with enhanced wall functions is used in computational fluid dynamics (CFD). Additionally, under steady conditions, the simple method has been used in the second order. The main focus is on power generation by an offshore CAWT, where the water is assumed to be calm, and the effects of possible waves have been disregarded. Consequently, sea level modeling has been abandoned. The boundary conditions for the inlet and outlet of the flow, as shown in Fig. 6, are chosen to be constant velocity (V = 8 m/s [11]) and outlet pressure (gauge pressure = 0), respectively. Furthermore, the upper, bottom, right, and left computational domain boundaries, which are far enough from the deflector geometry to not influence the characteristics of the flow passing through and around it, are selected as non-slippery walls. Additionally, the modeled deflector is also selected as a non-slippery wall. As demonstrated in Fig. 6, the computational domain is a cube. Moreover, the fluid domain presented in this article has the minimum dimensions so that the walls of the domain do not affect the characteristics of the flow passing through the deflector. This was done due to the reduction of the number of cells and also the simulation time.

### 3.2 Mesh independency

To reduce the cost of the numerical solution while achieving mesh independence and converging the results, the geometry has been meshed using 1,125,000 nodes, which is the smallest total number of nodes required for convergence. Table 4 displays the numerical modeling results of the average velocity at the deflector's upper surface for various cell counts.

**Table 4:** Mesh independency.

Row	Number of cells	Average velocity at the surface line of the deflector (m/s)
1	500,000	2.885
2	750,000	3.578
3	1,000,000	4.641
4	1,125,000	4.998
5	1,750,000	5
6	2,000,000	5

**Fig. 7:**
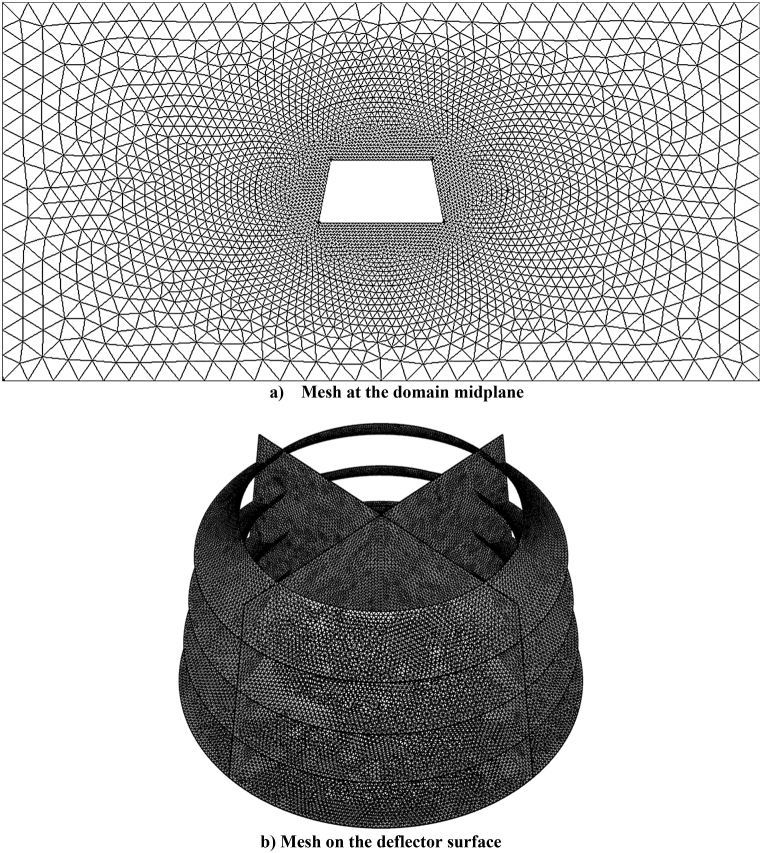
A graphical presentation of the meshing.

As the geometric model of the current research is three-dimensional and complex, an unstructured triangular grid has been used. According to the 3D model of the presented deflector and the mesh which includes 1,125,000 nodes, the simulation had been done for about 6 h to reach the steady condition by using 4 logical processors. Moreover, the target skewness had been set to be 0.7 with high smoothing to produce a high quality mesh. Fig. 7 displays a graphical representation of the meshing employed at the simulation. The computational domain mesh has been designed to have smaller cell dimensions near the deflector geometry, and gradually, as the distance from the deflector increases, the cell dimensions become larger. This is because the findings at areas far from the deflector are non-critical and require a lower level of calculation precision.

## 4 Analytical methods

### 4.1 DMST method

The DMST method is utilized for analyzing VAWTs. As depicted in Fig. 9, this method divides the turbine rotor into upwind and downwind halves. The streamlines path is also divided into several stream tubes along the flow direction, with the azimuth angle (Δθ) for segmentation corresponding to the stream tubes. When the wind strikes the rotor blades in the upwind direction, it loses some of its energy. Passing through the downwind blades also causes a decrease in wind velocity. Eventually, the flow exiting the rotor reaches its minimum velocity, which is known as wake velocity ($V_{w}$). The velocities at the upwind and downwind sections are $V_{au}$ and $V_{ad}$, respectively, and are influenced by the individual induction factors in the upwind and downwind strokes.

(8) $$V_{\infty }>V_{au}>V_{e}>V_{ad}>V_{w}$$

(9) $$V_{au}=a_{u}V_{\infty }$$

(10) $$V_{e}=V_{\infty }\left(2a_{u}-1\right)$$

(11) $$V_{ad}=a_{d}V_{e}$$

**Fig. 8:**
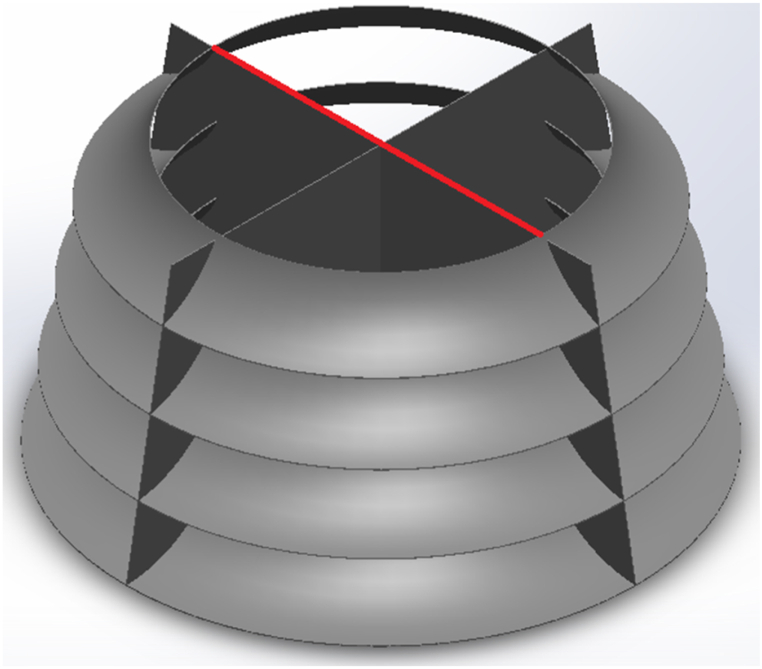
The surface line of the deflector (red line shown in the picture) where the average velocities for mesh independency is calculated for.

Fig. 8 depicts the DMST algorithm, which requires input variables such as blade length, rotor diameter, blade chord, airfoil type, rotational speed, and free wind speed to initiate the process. The blade is then discretized, and the tip speed ratio for each blade element is computed to begin the upwind procedure. The next step involves finding the upwind induction factor, which can be achieved through trial and error and applying default values for the induction factor. Once the appropriate induction factor is obtained, parameters such as relative speed and effective angle of attack for aerodynamic performance can be calculated. Equations (12), (13), (14), (15)) present normal velocity, tangential velocity, relative velocity, and the angle of attack.

(12) $$V_{n}=V_{a}\;\cos\;\theta$$

(13) $$V_{t}=r\omega -V_{a}\;\sin\;\theta$$

(14) $$V_{rel}=\sqrt{{\left(V_{n}\right)}^{2}+{\left(V_{t}\right)}^{2}}$$

(15) $$\alpha =\sin^{-1}\left(V_{n}/V_{rel}\right)$$

**Fig. 9:**
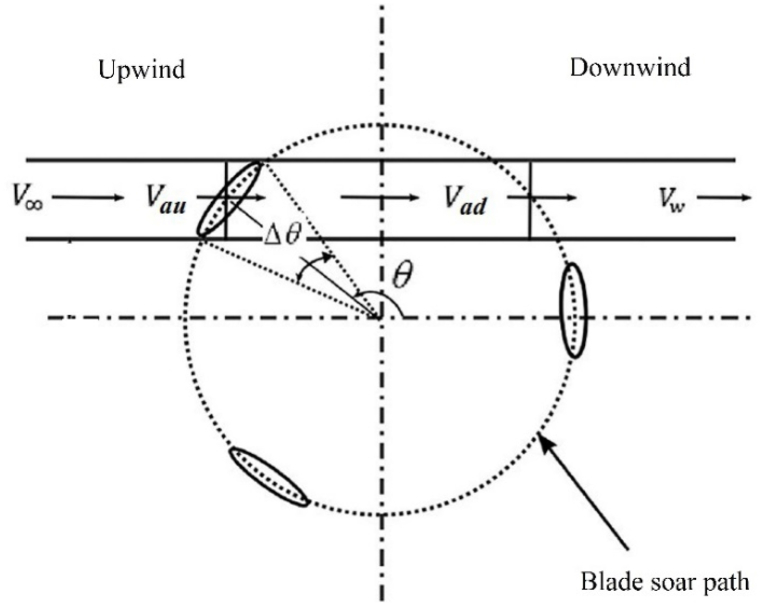
One typical Stream-tube used in the DMST method [22].

To improve the DMST approach, Prandtl's correction based on equation (11) was utilized. Blade tip vortices are caused by diverted flow from the high-pressure surface to the low-pressure surface near the blade's tip. Downwash flow and tip vortices reduce the angle of attack, resulting in a lower effective angle of attack than the apparent angle of attack. This phenomenon decreases aerodynamic performance. In this case, relationships 17 and 18 can be used to calculate the lift and drag coefficients, respectively.

(16) f=N/2((1−η)/(ηsin|α|))

(17) $$C_{L\left(3D\right)}=C_{L\left(2D\right)}\left(2/\pi \right)\cos^{-1}\left(e^{-f}\right)$$

(18) $$C_{D\left(ind\right)}=\left(C_{L\left(3D\right)}^{2}/\left(0.95\pi .AR\right)\right)$$

The normal and tangential force of the blade can be calculated by computing the relative velocity and angle of attack, similar to relations 19 to 22. The torque applied to the blade is then computed. The downwind stroke calculations follow the same pattern as the upwind stroke, but with $V_{e}$ replaced by $V_{\infty }$. After determining the induction factor using equations (23), (24)), this loop can be performed for each element of the blade and each azimuth angle until convergence occurs. It is worth noting that the induction factors in the DMST approach differ in upwind and downwind strokes and must be investigated independently.

(19) $$C_{n}=C_{L\left(3D\right)}\;\cos\;\alpha +C_{D}\;\sin\;\alpha$$

(20) $$C_{t}=C_{L\left(3D\right)}\;\sin\;\alpha -C_{D}\;\cos\;\alpha$$

(21) $$F_{N}\left(\theta \right)=\left(\frac{1}{2}C_{n}.\rho .c.\Delta h.V_{rel}^{2}\right)$$

(22) $$F_{T}\left(\theta \right)=\left(\frac{1}{2}C_{t}.\rho .c.\Delta h.V_{rel}^{2}\right)$$

(23) $$f=\frac{Bc}{8\pi r}\int_{-\pi /2}^{\pi /2}\left(C_{n}\frac{\cos\;\theta }{\left|\cos\;\theta \right|}-C_{t}\frac{\sin\;\theta }{\left|\cos\;\theta \right|\cos\;\delta }\right){\left(\frac{V_{rel}}{V_{a}}\right)}^{2}d\theta$$

(24) $$a_{new}=\pi /\left(f+\pi \right)$$

(25) $$Q\left(\theta \right)=r.F_{T}\left(\theta \right)$$

(26) $$\bar{Q}=\frac{N}{2\pi }\int Qd\theta$$

(27) $$C_{\bar{Q}}=\frac{\bar{Q}}{1/2.\rho .A.R.U_{\infty }^{2}}$$

(28) $$C_{P}=\lambda .C_{\bar{Q}}$$

Finally, the values of normal and tangential forces on each blade section can be integrated to calculate the total forces applied to the entire turbine. Equations (25), (26), (27), (28)) can be used to derive torque and power coefficient, where Q is one blade torque varying with azimuth angle, $\bar{Q}$ is the rotor average torque, $C_{\bar{Q}}$ is the average torque coefficient, and $C_{P}$ is the power coefficient. Therefore, Prandtl's correction factor was used in this method to consider the effect of blade tip vortices. Blade tip vortices have one of the most significant impacts on the turbine's aerodynamic performance. This factor causes changes in the effective angle of attack and the lift and drag coefficients, which are considered in relations 17 and 18. By incorporating these corrections, the performance of this method has been improved compared to the classical DMST approach.

### 4.2 BEM method

By combining the blade element theory and momentum theory, the BEM theory is derived. The blade of a wind turbine is divided into a limited number of independent elements, with each element generating a circular flow. The induction velocities in the axial and tangential directions for each element are obtained using the momentum theory. The blade element theory is then applied to calculate the aerodynamic forces acting on each element. By repeating the input conditions of the problem, the total aerodynamic forces acting on the blade can be determined for each condition. Using Bernoulli's law and assuming a certain number of elements on each blade, the thrust and torque on each element are calculated as follows:

(29) $$dT=\sigma \pi \rho \frac{U_{\infty }^{2\;}{\left(1-a\right)}^{2}}{\sin^{2}\;\varphi }C_{n}rdr$$

(30) $$dQ=\sigma \pi \rho \frac{U_{\infty }\left(1-a\right)\left(1+a^{\prime }\right)r\omega }{\sin\;\varphi \;\cos\;\varphi }C_{T}r^{2}dr$$

**Fig. 10:**
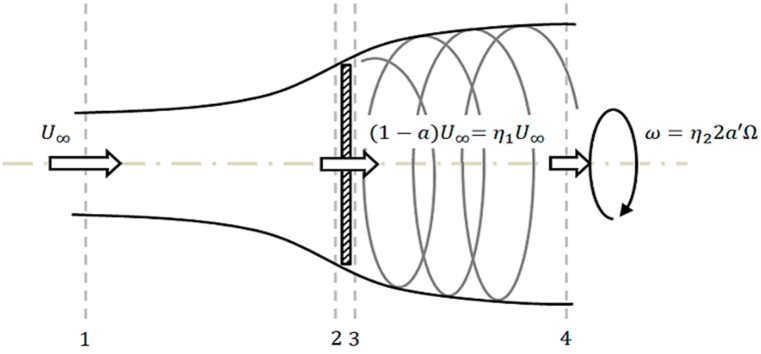
A control volume around a rotor with wake rotation [22].

Here, σ represents the solidity of the rotor, and a and $a^{\prime }$ are the axial and tangential induction factors, respectively. Fig. 10 illustrates the impact of axial and tangential induction factors (a and $a^{\prime }$) on flow velocity.

To improve the accuracy of problem-solving, the blade element theory incorporates Prandtl's loss factor and turbulent wake correction. When dealing with a rotor with a limited number of blades, the blade vortices differ significantly from those of a rotor with an infinite number of blades. The current relationships assume an infinite number of blades, but with the limited number of blades and the presence of vortices at the tip of each blade, force and momentum equations must be adjusted. Prandtl's loss factor accounts for the effect of the discontinuity of the rotor disk and includes tip and hub loss factors. It is defined as follows:

(31) $$F=\left[\frac{2}{\pi }\cos^{-1}\left(e^{-\frac{B}{2}\frac{R-r}{rsin\left(\emptyset \right)}}\right)\right]\left[\frac{2}{\pi }\cos^{-1}\left(e^{-\frac{B}{2}\frac{r-R_{hub}}{rsin\left(\emptyset \right)}}\right)\right]$$

The BEM method used in this research is an improvement over the classical method, as it considers the effects of blade tip loss and loss caused by the blade root (hub loss) [23,24]. However, if the axial induction factor exceeds 0.5, the velocity value at the output of the control volume becomes negative, rendering the momentum theory invalid and unable to provide an accurate prediction of the wind turbine thrust coefficient. Hence, corrections to the obtained relations are necessary. Several researchers have presented various relations, and the most accurate one is the Glauert [25] model, with a critical axial induction factor of 0.2, given below:

(32) $$C_{T}=4a\left(1-\frac{\left(5-3a\right)a}{4}\right)F$$

The thrust forces computed for each radial element throughout the blade span are equivalent to both the momentum theory and the blade element theory, resulting in the production of axial and tangential induction factors, as shown in the two equations below.

(33) $$a=1/\left(\frac{4F\;\sin^{2}\left(\emptyset \right)}{\sigma C_{n}}\right)$$

(34) $$a^{\prime }=1/\left(\frac{4Fsin\left(\emptyset \right)\cos\left(\emptyset \right)}{\sigma C_{t}}-1\right)$$

Equation (33) holds only if the axial induction factor is less than the critical value ($a<a_{c}$). If the axial induction factor exceeds the critical value ($a>a_{c}$), the value of the thrust coefficient must be determined using one of the various models proposed to correct the turbulent wake. Consequently, the value of the new axial induction factor is obtained by using the thrust equalization obtained for each radial element along the blade span by the momentum theory and the blade element theory. The modified value for the axial induction factor for the Glauert [25] model is as follows:

(35) $$a=\left(0.143\sqrt{0.0203-0.6427\left(0.889-C_{T}\right)}\right)/F$$

By specifying the modified axial induction factor, the values of the axial induction factor for each element along the blade span can be calculated using an iterative pattern. Once the axial and tangential induction factors are determined, it is possible to calculate the thrust, momentum, and power.

## 5 Analytical methods validation

### 5.1 DMST method

**Fig. 11:**
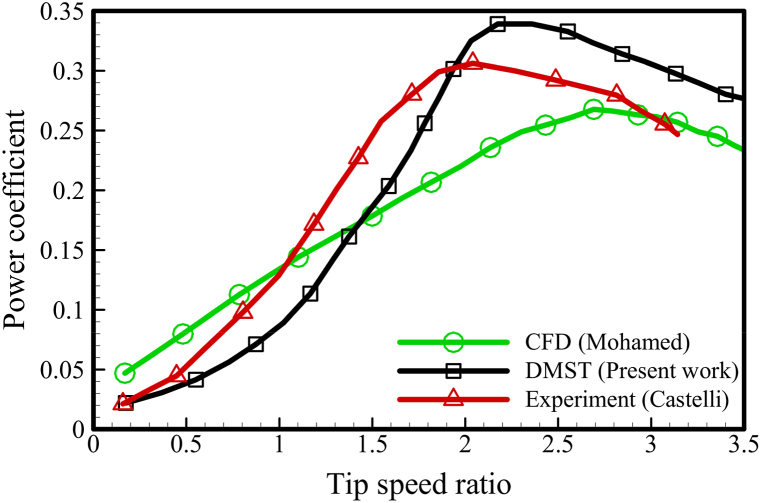
DMST method validation for VAWT.

To validate the DMST procedure, an H-type turbine with the geometrical characteristics specified in Table 5 was used. The power coefficient obtained from the proposed method and implemented code was compared to both experimental data in Ref. [26] and numerical analysis in Ref. [27]. The horizontal and vertical axes in Fig. 11 represent the dimensionless parameters of the blade tip speed ratio (TSR) and the turbine power coefficient ($C_{P}$), respectively. Based on this figure, the DMST approach described in this study accurately estimated the baseline turbine's power coefficient within a 14 percent relative error, which is acceptable for vertical axis wind turbines (VAWTs) that experience separation and complex wake interactions.

**Table 5:** Characteristics of basic VAWT.

Parameter	Value
Airfoil type	NACA 0021
Number of blades	3
Blade chord	0.08 m
Blade height	1.5 m
Rotor diameter	1 m

### 5.2 BEM method

**Fig. 12:**
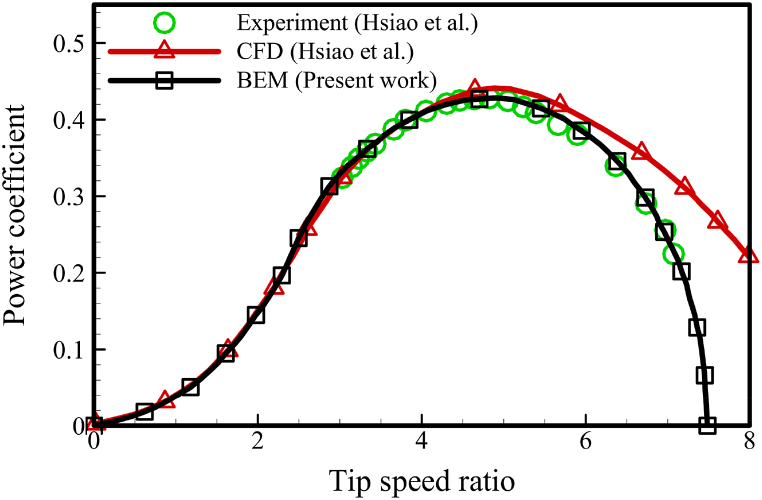
BEM method validation for HAWT.

To validate the MATLAB BEM code, the reference turbine and blade geometry from Hsiao et al.'s research are used. The resulting data will be compared with experimental, numerical, and analytical results from the same study [28]. Hsiao et al. studied three different types of horizontal axis wind turbines with a diameter of 0.72 m. Fig. 12 shows a comparison of the power coefficient results obtained from the BEM code designed in this work with the experimental and CFD results of ref. [28]. The results obtained from the BEM method are in good agreement with the experimental data and the numerical solution of Hsiao et al. This can be attributed to the consideration of the mutual effects of the flow and the turbine rotor, including the effect of the wake formed behind the rotor, as well as the Prandtl's correction factor for the blade tip and root losses. Table 6 provides complete information on the optimum blade shape of Hsiao et al., which is used for validation.

**Table 6:** Information on the optimum blade shape of Hsiao et al. [27].

Characteristic	Value
Rated power (W)	50
Rated wind speed (m/s)	10
Designed tip speed ratio	5
Number of blades	3
Designed angle of attack (deg)	5.5
Airfoil type	NACA 4418

## 6 Results

### 6.1 Effect of deflector on wind velocity

**Fig. 13:**
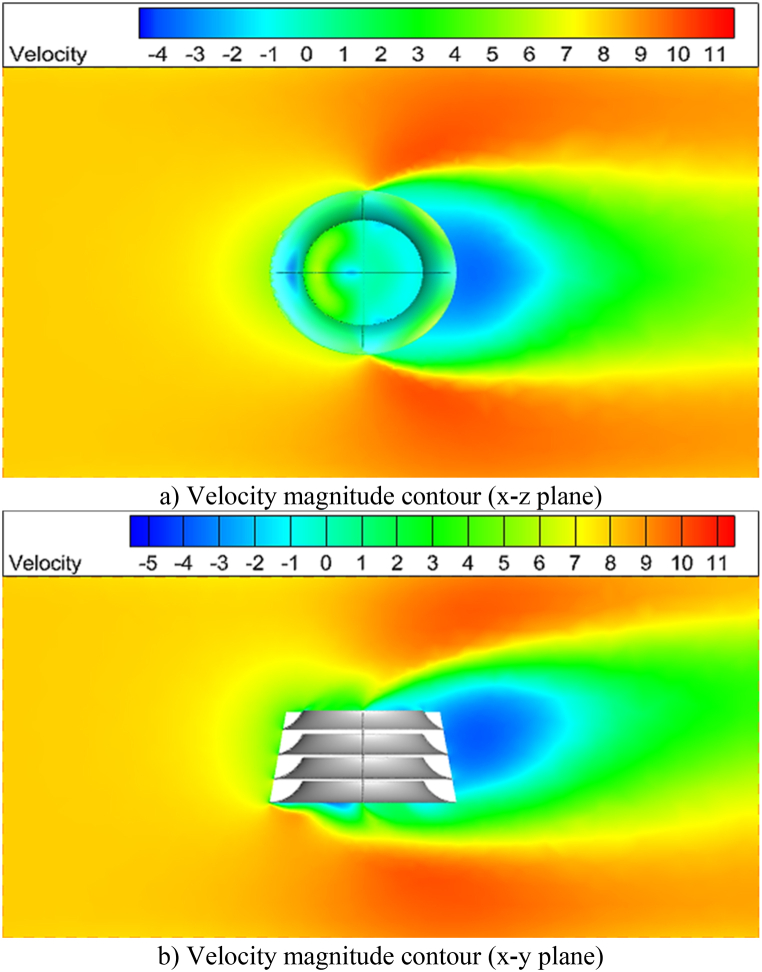
Velocity magnitude contours by considering inlet velocity = 8 m/s.

Fig. 13 depicts velocity magnitude contours in the x-z and z-y planes, as well as streamlines at the x-y midplane. These figures show that a flow with a velocity of 8 m/s is incident on the deflector from the left side and changes direction. As a result of this shift in direction, a large portion of the flow exits from the upper area of the deflector. This component of the flow velocity (in the Y direction) provides torque and power by striking the HAWT blades. The velocity contours depict that the average flow velocity at the upper edge of the deflector is roughly 5 m/s. Moreover, some of the flow exits from the deflector's lower edge. Since the deflector is situated on the surface of the sea, the flow flowing out of the bottom of the deflector is expected to disappear, strengthening the top flow component.

**Fig. 14:**
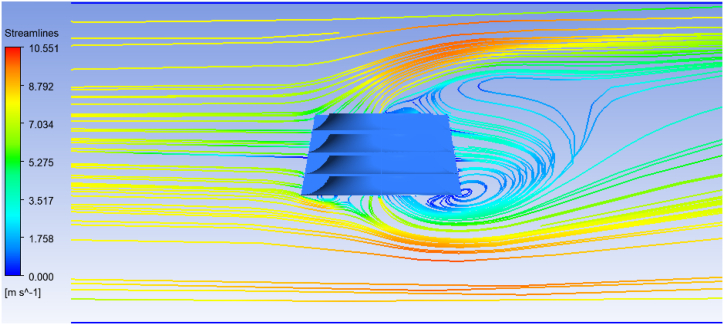
Streamlines at x-y midplane by considering inlet velocity = 8 m/s.

Fig. 14 illustrates the flow streamlines as they pass through the deflector. When the flow hits the deflector at its upper and lower edges, it changes direction and velocity. However, at the rear of the deflector, the flow velocity has decreased, and the direction has changed, resulting in a vortex flow.

### 6.2 CAWT performance

In the preceding section, we explored the effect of the designed deflector on the wind. The deflector forces a portion of the flow to change direction and escape vertically above it. By mounting the CAWT on this deflector, the HAWT blades (which serve as the VAWT's supporting arms) are placed on top of the deflector. As a result, the vertical flow emerging from the top of the deflector is projected to collide with these blades, generating additional power in the HAWT blades.

**Fig. 15:**
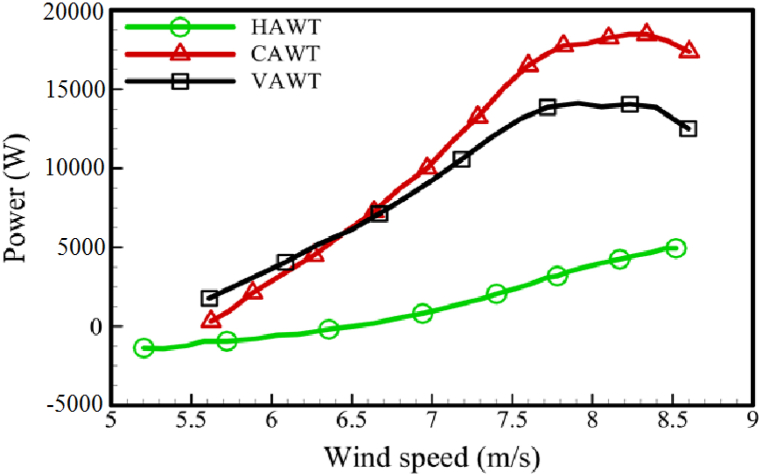
Power generated by HAWT, VAWT, and CAWT.

Fig. 15 illustrates the power generated by the CAWT, VAWT, and HAWT individually. In this picture, the horizontal axis represents the horizontal wind speed ($V_{\infty }$). When comparing the graphs of horizontal and vertical axis turbine blades, it is evident that VAWT blades generate the majority of the power. The CAWT power curve is the sum of the previous two curves. It can be observed that the CAWT produces 19 KW of power when the wind speed is between 8 and 8.5 m/s (the working range of the assumed wind turbine). At the same wind speed, this value for a VAWT is around 14 KW. Therefore, we conclude that a CAWT turbine with a deflector generates approximately 35 % more power than a VAWT turbine.

**Fig. 16:**
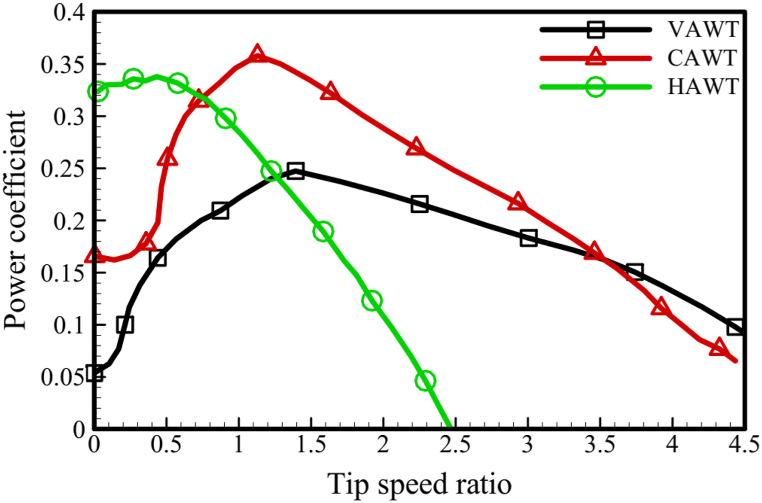
Power coefficient of HAWT, VAWT, and CAWT.

The power coefficient curve can also be utilized to assess the efficiency of turbines. Fig. 16 depicts these curves. The vertical axis in this figure represents the turbine's power coefficient, which is calculated by dividing the turbine's generated power by the power of the wind passing through the turbine's cross-section. The horizontal axis in this figure represents the blade tip speed ratio (TSR), which is calculated by dividing the blade tip's rotational speed by the wind speed. It should be noted that the non-dimension power ($C_{P}$) of the VAWT and HAWT blades differ in this figure. This is due to the different input wind speeds and differential cross-sections of the turbines. However, since the primary objective is to evaluate the CAWT, the dimensionality of this turbine's power coefficient has been based on the horizontal wind speed ($V_{\infty }$) and the cross-sectional area of the VAWT turbine (H×D). Fig. 16 shows that the maximum power coefficient for each of the VAWT and HAWT blades is 0.246 at TSR = 1.5 and 0.336 at TSR = 0.5, respectively. Finally, at TSR = 1.2, the maximum power coefficient of the CAWT is 0.356. Therefore, under the same conditions, the CAWT turbine is expected to have a 45 % higher efficiency than the VAWT turbine. Table 7 summarizes the performance of each turbine's blades.

**Table 7:** Summary of CAWT and VAWT performance.

	Max Power (KW)	TSR for $P_{\max}$	Max Power Coefficient	TSR for ${CP}_{\max}$
**VAWT**	14	2.9	0.246	1.3
**CAWT**	19	2.8	0.356	1.2
**Improvement**	5 (35 %)	–	0.11 (45 %)	–

## 7 Conclusion

The CAWT turbine is considered a novel wind turbine that is more efficient than the VAWT type, but creating vertical flow is challenging. Previous methods include tall buildings or inclined plates, but this study uses a CAWT turbine with a special deflector on the sea surface. The turbine benefits from wind from all directions and is away from sea turbulence. The turbine's output power is determined by checking the flow through the deflector and evaluating the rotor using DMST and BEM methods. These methods consider effects such as blade tip loss, root loss, and wake effect. The simulation shows that horizontal wind hits the VAWT blades while the vertically deflected flow passes through the HAWT blades. One significant advantage of the analytical methods used in this research is their potential for development for turbines with different specifications and reduced computational time. According to the flow simulation in this deflector, if the incoming horizontal wind flow is equal to 8 m/s, the average speed of the vertically deflected flow is about 5 m/s. Therefore, horizontal wind with a speed of 8 m/s hits the VAWT blades, while vertical wind flow with a speed of 5 m/s passes through the HAWT blades.

The results of this evaluation show that the CAWT can achieve a maximum power of 19 KW with a horizontal wind speed of 8.4 m/s. Furthermore, an examination of the power coefficient curve revealed that the turbine's efficiency is 35.6 percent at TSR = 3.2. When comparing the CAWT to the VAWT under the same conditions, the power and power coefficient obtained from the CAWT turbine are 35 percent and 45 percent higher, respectively. However, the study's limitations include sea turbulence, albeit small, and the creation of unsteady flow. Therefore, it is recommended to develop the method to consider these cases and optimize it for future work.

**Table:** Nomenclature

Symbol	Parameter
a	Axial Induction factor
$a^{\prime }$	Tangential Induction factor
$V_{\infty }$	Wind speed
$V_{e}$	Induced equilibrium velocity
$V_{n}$	Normal velocity
$V_{t}$	Tangential velocity along the blade chord
$V_{s}$	Tangential velocity along the blade length
$V_{rel}$	Relative velocity
$V_{z}$	Velocity along the rotor axis
$V_{a}$	Axial induction velocity
$C_{n}$	Normal force coefficient
$C_{t}$	Tangential force coefficient
f	Blade function
N	Normal force
T	Tangential force
λ	Tip speed ratio
ω	Rotating speed
$\tau_{ij}$	Reynolds stress
∂P/∂x	Pressure gradient
AR	Aspect ratio
W	Weight force
m	Total mass
ρ	Air density
S	Deflector bottom base area
$\dot{m}$	Mass flow rate
**u**	Average velocity
Q	Local torque
$\bar{Q}$	Mean torque
$C_{\bar{Q}}$	Mean torque coefficient
$C_{P}$	Power coefficient
$C_{L}$	Lift coefficient
$C_{D}$	Drag coefficient
L	Lift
D	Drag
r	The local radius of the rotor
Δz	Blade element height
B	Number of Blades
c	Blade chord
A	Swept area
α	Angle of attack
η	Non-dimensional blade length
ρ	Air Density
$\rho_{w}$	Water density
σ	Rotor solidity
**F**	Prandtl's loss factor
**B**	Buoyancy force
∀	Deflector volume

## Declaration of competing interest

The authors declare that they have no known competing financial interests or personal relationships that could have appeared to influence the work reported in this paper.
